# Pertinence of Constraint-Induced Movement Therapy in Neurological Rehabilitation: A Scoping Review

**DOI:** 10.7759/cureus.45192

**Published:** 2023-09-13

**Authors:** Purva Gulrandhe, Sourya Acharya, Maharshi Patel, Samarth Shukla, Sunil Kumar

**Affiliations:** 1 Department of Physiotherapy, Ravi Nair Physiotherapy College, Datta Meghe Institute of Higher Education and Research, Wardha, IND; 2 Department of Medicine, Jawaharlal Nehru Medical College, Datta Meghe Institute of Higher Education and Research, Wardha, IND; 3 Department of Pathology, Jawaharlal Nehru Medical College, Datta Meghe Institute of Higher Education and Research, Wardha, IND

**Keywords:** rehabilitation, physical therapy, neurorehabilitation, cerebral palsy, stroke, constraint-induced movement therapy

## Abstract

Constraint-induced movement therapy (CIMT) is a neurorehabilitation technique that aims to restore motor function in patients with central nervous system injuries. Based on behavioral research conducted, CIMT has been found effective in restoring motor function in various conditions including stroke, cerebral palsy, traumatic brain injury (TBI), and more. The therapy combines neurological and behavioral mechanisms to induce neuroplastic changes and overcome learned nonuse. Modified CIMT (mCIMT) is a variant that focuses on sensorimotor functioning in the affected limb. This review summarizes studies on CIMT and mCIMT, with a focus on stroke, cerebral palsy, and other conditions. Results show that CIMT and mCIMT demonstrate significant improvements in motor function and quality of life. The studies underscore the importance of long-term research, comparative or combined therapies, and exploration of less-studied conditions like multiple sclerosis (MS) and brachial plexus injury. Overall, CIMT and mCIMT hold promise for neurorehabilitation, emphasizing the need for further investigation to enhance their effectiveness and application.

## Introduction and background

Constraint-induced movement therapy (CIMT), a neurorehabilitation technique based on behavior, was investigated through preclinical large animal primate studies [1]. It has been found to significantly restore motor function in patients with a variety of injuries to the central nervous system. CIMT essentially incorporates two mechanisms: a neurological mechanism for enabling neuroplastic alteration and a behavioral mechanism for overcoming learned nonuse [1]. Inhibiting maladaptive plasticity in patients with an acquired or congenital unilateral hemispheric pathology is a central objective of CIMT. This approach involves restraining the unaffected limb while implementing an intensive motor activity training program focused on the paretic limb, aiming to improve or restore motor function. The time necessary to carry out the treatment plan given by Taub et al. was six hours of supervised training of the affected extremity every day, 90% of the time with the restrained non-affected limb, and conducted for fourteen consecutive days [2]. CIMT has demonstrated improvements in restoring the paretic limb's functioning, in the functional range of motion, and in lowering muscle tone, leading to a better quality of life [2].

Strong evidence supports the use of modified mCIMT for improving the use and restoration of sensorimotor functioning in the paretic limb [3]. The three main components of mCIMT are (1) repetitive training of paretic limbs for a prolonged period; (2) a "transfer package"; and (3) limited usage of the non-paretic limb, which forces the person to use the paretic limb. mCIMT had a substantial impact on motor control and daily activity function when compared to conventional rehabilitation treatment [4].

## Review

Method 

A scoping review technique was employed to get a summary of the literature available on the application of CIMT and mCIMT in stroke, cerebral palsy (CP), and other conditions. 

Data Source and Search Strategy

From 2017 to December 2022, an electronic search was conducted in CINAHL, Pedro, Medline, Embase, PubMed, Scopus, and Google Scholar. These resources were chosen because they provide an extensive overview of healthcare-related fields, including physical rehabilitation. Systematic reference list checks and Google Scholar citation monitoring of retrieved papers were performed to make sure that all necessary research was gathered. As search terms, we used CIMT, stroke, CP, modified CIMT, and physical rehabilitation.

Inclusion and Exclusion Criteria

The inclusion criteria for articles in this review were based on two key factors: availability of a full-text copy and relevance to CIMT or mCIMT in the context of conditions like stroke, CP, or other relevant conditions. The exclusion criteria included studies with incomplete data, those disqualified due to errors or irregularities, publications outside the specified date range, articles not in English, and duplicate publications.

Charting Extraction

A form for the extraction of data was created. Authors, country of origin, publication year, source or type of publication, and methodology, which included the use of terminology, description, and procedure, including theoretical context and basis, were used to organize data.

Data Synthesis and Analysis

**Figure 1:**
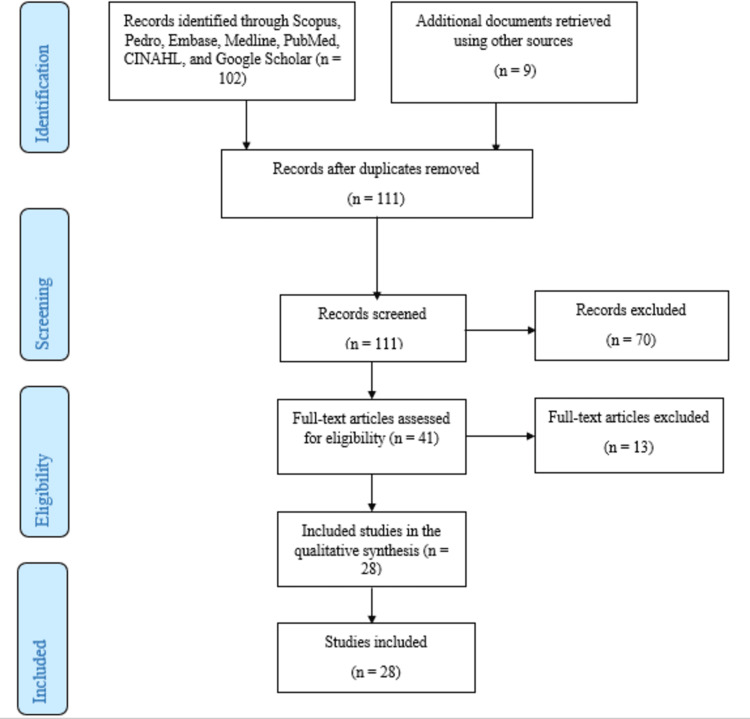
PRISMA flowchart showing the data selection process PRISMA, Preferred Reporting Items for Systematic Reviews and Meta-Analyses

Following the charting of data, information was arranged according to the year of publication, published sources, methodology, and conclusion. The PRISMA (Preferred Reporting Items for Systematic Reviews and Meta-Analyses) flow chart on the existing literature on CIMT and mCIMT is given in Figure 1.

Result

The electronic data search produced a total of 111 articles. The PRISMA flowchart outlines the step-by-step process for article selection. Scopus, CINAHL, Pedro, Embase, Medline, PubMed, and Google Scholar were among the electronic databases used. Records checked at the first level of screening were 111, while 70 articles were eliminated. 41 articles qualified based on inclusion criteria, but 13 were disqualified due to errors, duplications, and other irregularities. The study included 28 articles in total out of which 16 articles were based on stroke, eight articles on CP, and four articles on other conditions. In all, 934 patients were studied for the review. Patients with 479 strokes, 368 cerebral palsies, and 87 other conditions were among those included in the study.

Stroke

Stroke is the second most frequent cause of mortality and one of the leading causes of acquired disability [5]. Each year, 16.9 million individuals worldwide experience their first stroke, leading to around 33 million patients with stroke and 5.9 million deaths due to stroke. Around 80% of these individuals have severe upper limb motor deficits that seriously impede their capacity to conduct activities of daily living and engage in social activities [5]. mCIMT was designed to provide post-stroke patients with more functional use of their affected limb, reverse learned nonuse, and enhance motor control and manual dexterity. High amounts of evidence support the recovery of the limb after stroke with mCIMT [3]. Table 1 below summarizes research that has been conducted and highlights the effects of CIMT over other treatment methods.

**Table 1:** Summary of studies on stroke CIMT, constraint-induced movement therapy; mCIMT, modified constraint-induced movement therapy

Author name and year	Patient population	Study group	Intervention	Conclusion
Mary Ann Smith et al. 2020 [6]	Chronic Hemiparesis	28	Modified constrained-induced movement therapy using telehealth	It was found to be effective and improve functional ability.
Cathryn R. Baldwin et al. 2018 [7]	Stroke	19	CIMT	It was used in the Australian community rehabilitation setting making it a beneficial and cost-effective approach.
M M Marándola et al. 2020 [8]	Ischemic stroke with hemineglect	30	mCIMT vs. conventional therapy	mCMIT may be clinically used in hemineglect to improve symptoms.
Larissa Salgado Oliveira Rocha et al. 2021 [2]	Chronic hemiparetic	30	CIMT vs. conventional therapy	Both protocols proved substantial improvement in quality of life and functionality.
Han-Chin Hsieh et al. 2021 [9]	Hemiplegia	35	Kinesio taping and mCIMT	Motor performance was improved when the combination of Kinesio taping and mCIMT was provided.
Gitendra Uswatte et al. 2018 [10]	Severe upper-extremity hemiparesis	21	An expanded form of CIMT (eCIMT)	eCIMT proved to be effective.
Mohammad Nasb et al. 2019 [11]	Stroke	64	Botulinum-A toxin-mCIMT (BTX-mCIMT) vs BTX with high-dose conventional therapy	BTX-mCIMT showed higher benefits than BTX with high-dose conventional therapy on motor control and activities of daily living.
Hee Kim et al. 2018 [12]	Stroke	14	The combined therapy of mCIMT and mental practice	Combined therapy has a higher benefit and the effect of mental practice should be considered.
Arlette Doussoulin et al. 2018 [13]	Stroke more than six months	36	Group therapy, compared with individual mCIMT	Both approaches were effective but more improvement was seen in group therapy.
Gitendra Uswatte et al. 2021 [14]	≥1-year post-stroke with chronic upper-arm extremity hemiparesis	24	Tele-health CIMT and in-lab CIMT	Tele-AutoCITE has immediate benefits over in-lab CIMT.
Rena Chamudot et al. 2018 [15]	Hemiplegia	33	mCIMT vs. bimanual therapy	Both approaches are equally beneficial.
Sun Ho Kim 2021 [16]	Chronic stroke patients	30	Dual transcranial direct current stimulation and mCIMT	It has shown therapeutic efficiency.
Nuray Alaca et al. 2022 [17]	Chronic stroke	40	Proprioceptive-based training and modified CIMT	Both the therapies applied in combination showed a greater effect.
Saleh M Aloraini 2022 [18]	Stroke	38	CIMT for the lower extremity (CIMT-LE)	The intervention showed clinical improvement.
Dae-Hyouk Bang et al. 2018 [19]	Early stroke	24	mCIMT modified by adding trunk restraint (TR)	A combination of both interventions is more effective.
Anne Stark et al. 2019 [20]	Chronic stroke	13	homeCIMT	It may help in improving the home program.

Cerebral palsy

CP is the most common physical disability among children, which affects one to three out of every 1000 live births [21]. 38% of incidences involve unilateral CP [21]. These children have motor and sensory deficits on either side of the body, which are frequently more significant in the upper limb. Their involvement in society and quality of life is impacted by these sensorimotor deficits, which often result in reduced capacity to perform everyday tasks. While motor execution is the major focus of mCIMT, research has demonstrated that children with unilateral CP also exhibit abnormalities in motor representations related to movement planning. Therefore, therapies that concentrate on motor representations may improve learning and recovery even more [21]. Table 2 below provides a summary of studies conducted on CP patients with their findings.

**Table 2:** Summary of studies on CP CIMT, constraint-induced movement therapy; mCIMT, modified constraint-induced movement therapy; CP, cerebral palsy; BIT, bimanual training

Author name and year	Patient population	Study group	Intervention	Conclusion
Sharon Landesman Ramey et al. 2021 [22]	Hemiparetic CP	118	CIMT	CIMT was found to be effective when given in high doses.
Cristina Simon-Martinez et al. 2020 [21]	Unilateral CP	36	mCIMT vs. action-observation training	There was limited effectiveness of action-observation training to mCIMT.
Hasan Bingöl et al. 2022 [23]	Hemiplegic CP	32	mCIMT vs. BIT	mCIMT proved to be more beneficial.
Young Sub Hwang et al. 2020 [24]	Unilateral CP	24	mCIMT and a continuous restraint	The intervention was found to be useful in real-world practice.
Pauline M Christmas et al. 2018 [25]	Hemiplegic CP	62	Caregiver-directed CIMT	It was found to be effective and clinical improvements were seen.
Mamoona Tasleem Afzal et al. 2022 [26]	Hemiplegic CP	40	Classic CIMT vs. its modified form	Both interventions are found to be equally effective.
T Tawonsawatruk et al. 2022 [27]	CP	19	CIMT vs. operative methods	Operative methods showed more improvement.
Ann-Christin Eliasson et al. 2018 [28]	Unilateral CP	37	Baby-CIMT vs. massage	CIMT was more beneficial than massage.

Other neurological conditions

In addition to potential sensory deficits, brachial plexus traction during labor and delivery produces a brachial plexus birth injury, which results in flaccid paresis or upper limb paralysis. Physiotherapy and occupational therapy are the cornerstones of treatment for brachial plexus birth injury in children, encouraging both passive and active movement of the affected upper limb to improve neuromuscular function and minimize problems caused by immobility [29]. CIMT is a more successful strategy in improving upper-limb function resulting from brachial plexus palsy in children [30].

Multiple sclerosis (MS) is an autoimmune disease of the central nervous system. MS can cause debilitating hemiparesis, among other clinical consequences. Multiple studies have indicated that various physical rehabilitation treatments can improve poor motor function in MS patients, albeit the treatment benefits are generally small and their long-term retention and transfer to spontaneous movement in real-world conditions is hardly evaluated. In individuals with hemiparetic MS, CIMT significantly improved real-world extremity function and caused white matter alterations [1]. Table 3 gives a summary of studies conducted on brachial plexus palsy and MS with their conclusion.

**Table 3:** Summary of studies on other conditions CIMT, constraint-induced movement therapy; mCIMT, modified constraint-induced movement therapy; MS, multiple sclerosis; CAM, complementary and alternative medicine

Author name and year	Patient population	Study group	Intervention	Conclusion
Alessandro de Sire et al. 2019 [31]	MS	20	CIMT	It was found to be safe and effective.
Julie M Werner et al. 2020 [30]	Neonatal brachial plexus palsy	21	CIMT	The approach favored the use of conventional therapy.
Beyhan Eren et al. 2020 [32]	Perinatal brachial plexus palsy	26	mCIMT	It has been shown to improve the range of motion and improve functionality.
Ameen Barghi et al. 2018 [1]	MS	20	CIMT or CAM	CIMT showed white matter changes along with improvement.

Discussion

This review includes experimental studies. The studies are emphasizing CIMT and mCIMT on patients with brachial plexus injury, MS, CP, and stroke. When compared to other therapy like conventional therapies, bimanual therapy action-observation training, and massage, CIMT is successful in the majority of cases. There have been a few studies that have employed a combination of treatments that have proven to be highly beneficial. Most of the studies are on stroke patients but more long-term studies need to be conducted. In the case of CP patients, there is a need for conducting studies on comparative or combination therapy. Few literatures are available for patients with MS and brachial plexus injury.

In their study, Davide Corbetta et al. concluded that CIMT is a multimodal strategy in which limiting the use of the non-paretic extremity is combined with the increased activity that is capacity-appropriate [33]. They discovered that while CIMT was related to modest improvements in motor function and impairment, these advantages did not significantly reduce disability [33]. According to D M Morris et al., the CIMT involves several tasks and subtasks that work in unison to achieve the favorable results observed by treatment package subjects [34]. The CIMT treatment technique offers a significant paradigm change for traditional physical rehabilitation, as it is used in the UAB research lab and clinic [34]. The functional recovery and more conventional compensating techniques are distinct from the CIMT treatment approach in several significant respects. As there are fewer current studies available, there is a need for research on patients with traumatic brain injury (TBI). A 2012 study by Veronica Cimolin et al. revealed that CIMT can enhance upper extremity function and movement efficiency in children who have suffered TBI [35]. The integration of upper limb kinematics and clinical outcomes was shown to be essential in identifying the benefits of CIMT training [35].

## Conclusions

Both CIMT and mCIMT are beneficial in treating patients and improving the functioning of motor control and quality of life. The majority of studies are on stroke patients, although long-term research is still needed. Studies on alternative or combined therapies are required for patients with CP. For patients with MS and brachial plexus damage, there is a dearth of literature. This increases the potential for performing experimental studies in this population.
